# The effect of synchronised metronome training: A case study in a single leg, below knee Paralympic sprinter

**DOI:** 10.4102/ajod.v7i0.367

**Published:** 2018-05-23

**Authors:** Barry S. Andrews, Elizabeth S. Bressan

**Affiliations:** 1 Department of Sport, Recreation, and Exercise Science, University of the Western Cape, South Africa; 2 Centre for Human Performance Science, Stellenbosch University, South Africa

## Abstract

**Background:**

To optimise sprint performance, one needs to understand how motor control affects motor performance. Researchers have proposed that the Dynamic Systems Theory be adopted for explaining motor performance, skill acquisition and the development of pedagogical methods. Within this theory, the individual is seen as a complex system that functions as the interaction of many sub-systems. Entrained movements would be characterised by optimal sequencing, timing and grading of muscle activation. One of the identified control parameters for running is the rhythm in the coordination pattern.

**Objectives:**

The objectives of this study were twofold: firstly to investigate whether 6 weeks of timing and rhythmicity training using the computer-based Interactive Metronome™ (IM™) system improves motor timing and rhythmicity, and secondly to investigate whether such effects of IM™ influence the kinematic variables of a sprint.

**Methods:**

This study followed a semi-quantitative analysis case study approach using a Paralympic sprinter with a single below knee amputation participated in this study. Data for acceleration and maximal running velocity phases were collected using video recorders.

**Results and conclusions:**

As found by previous research, the IM™ programme improved the motor timing and rhythmicity of the athlete. However, in contrast to previous research, only minimal improvements, non-significant improvements, were seen in the actual motor performance. This athlete was an older more established athlete and it is therefore recommended that these types of programmes should be followed by young participants in the more fundamental phases of their movement development, to show best results.

## Introduction

In modern athletics, top-level competitors sprint all distances from 100 m to 400 m. The sprinting action requires fast reaction time, exceptional acceleration and an efficient running style (Carr 1991). For analysis purposes, the sprint has been divided into three phases by Mann and Sprague (1983), namely the acceleration phase, the maximal running velocity phase and the deceleration phase. The acceleration and maximal running velocity phases are said to be the main determinants of sprint performance, with the deceleration phase having a lesser effect on the overall sprint performance (Mann & Herman 1985). Each of these phases has unique kinematic characteristics that can optimise the performance during that specific phase (i.e. stride length and stride frequency) (Hay 1978; Hay & Reid 1988). Although the kinematics are similar within the phases, they are not identical during all phases and need to be evaluated in isolation (Hay 1993).

To better understand how to optimise sprint performance, it is important to understand how motor control affects motor performance. As advocates of an ecological approach for the understanding of motor control, Haywood and Getchell (2009) proposed that the Dynamic Systems Theory be adopted for explaining motor performance, skill acquisition and the development of pedagogical methods. Within this theory, the individual is regarded as a complex system that functions within the interactions of many sub-systems. These interactions ‘self-organise’ according to a functional goal or intention in a particular environmental context and in relation to a variety of constraints that are relevant to a particular situation (Davids, Button & Bennett 2008).

**FIGURE 1:**
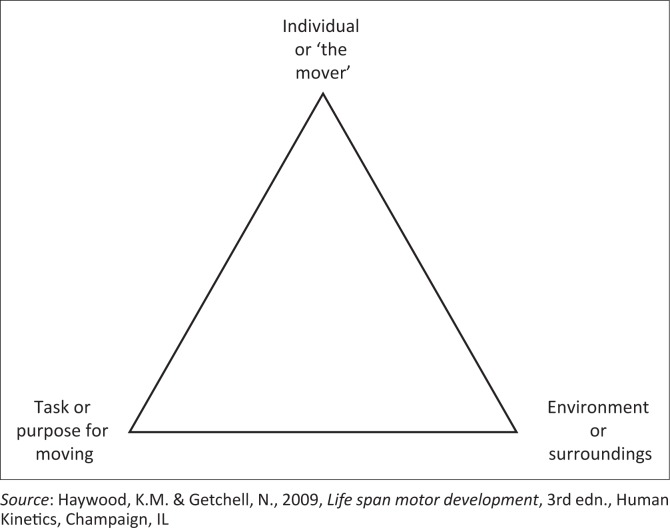
Newell’s model of the constraints that interact to shape motor performance.

Researchers investigating motor control and learning of sport skills often follow Newell’s model (Figure 1). This model identifies three kinds of constraints that influence the organisation of movement performance. These include individual constraints, task constraints and environmental constraints (Haywood & Getchell 2009). Following this model for athletes with physical disabilities (Andrews 2014), their individual constraints, which can be either structural or functional constraints, coupled with their goal (to sprint as fast as possible), need to be ‘self-organised’ for their ultimate success.

Proficiency in this ‘self-organisation’ process has been attributed to the development of fundamental coordinative structures. These structures allow the individual to adapt their movement patterns to achieve goals within the variety of constraints that impact on the particular situation (Davids et al. 2008; Magill 2003).

Chow et al. (2008) advocated the use of Newell’s model of three stages of motor learning to understand the progression from novice to expert. They linked these stages of motor skill learning to the progressive organisation and reorganisation of coordinative structures in response to changing constraints in movement performance situations. Davids et al. (2008) described this process as the development of coordination in which learning and practise lead to strengthened connections among the coordinative structures involved in task goal achievement. As the coordination patterns increase in stability, the individual is more able to adapt to the changing constraints of tasks and environments. Shumway-Cook and Woollacott (2007) described stable states as ‘well entrained systems’, referring to the reliability with which coordinated movement could be dynamically organised despite challenging performance circumstances. Well entrained movements would be characterised by the optimal sequencing, timing and grading of muscle activation patterns.

Motor control and timing are linked. The goal of good timing is the optimal synchronisation of synergies (Salman 2002). The running style of an elite sprinter is an example of a well entrained muscle activation pattern, as it is the manner in which these muscles are activated which gives the athlete their running style. Furthermore, running style could be described as a stable system in which the sequencing, timing and grading of muscle activation associate in as attractor well that is resistant to disruption. Practice activities that focus on optimising the sequencing, timing and grading of activation during sprint performance would be of interest to coaches and sprinters, if engaging in these activities produced an improvement in achieving the ultimate goal of sprinting – speed. One aspect of timing that has been identified as a control parameter for running is the rhythm entrained in the coordination pattern (Miller 2011). Rhythm was identified as a distinguishing characteristic of skilful performance and has been acknowledged to be a special type of timing that underlies the acquisition and performance of motor skills (Ben-Pazi, Kukke & Sanger 2007; Derri et al. 2001). According to Mastrokalou and Hatziharistos (2007), rhythmic ability is based on an internal representation of time that affects the way in which movements are performed. Hansen (2008) highlighted rhythm as a critical parameter in sprint performance. He explained that the optimal combination of stride frequency and stride length must be coordinated precisely during the different phases of a sprint. Furthermore, inappropriate rhythm in any given phase could result in muscle tightness, over-striding, premature depletion of energy and many other performance limiters (Hansen 2008).

Rhythm training programmes usually challenge individuals to synchronise their movements to an external rhythmic stimulus (Zachopoulou et al. 2003). Synchronisation has been improved in some individuals as a result of participation in a rhythm training programme (Greenspan 2002).

Thus, this study aimed to investigate whether 6 weeks of timing and rhythmicity training by means of synchronised metronome training (SMT) improves motor timing and rhythmicity. Furthermore, this study aimed to investigate whether such effects of SMT influence the kinematic variables of the sprint.

## Materials and methods

### Research design and sample

A semi-quantitative analysis case study approach was used for this study (Bartlett 2007). An elite male Paralympic sprinter with a single below-knee amputation participated in this study.

### Procedure

**FIGURE 2:**
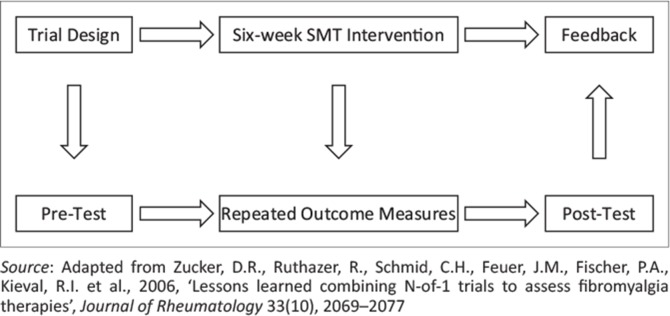
Scheme for the research design followed.

Kinematic data were gathered during the ‘Train to Compete’ stage of the periodisation plan within the athlete’s typical training regime (Bhambhani & Higgs 2011). Data from two phases of the sprint, the initial acceleration phase and the maximal running velocity phase, were collected using video recorders, aligned perpendicular to the sprint. These data were then calculated and analysed using video software (Dartfish ProSuite version 4.0.9.0). Following this, 6 weeks of timing and rhythmic training by means of SMT intervention was completed. Once this training was completed, kinematic investigation of the acceleration and maximal running velocity phases was gathered again, in order to see the potential effects of the SMT training (Figure 2).

This study used a very specific form of SMT training: The Interactive Metronome™ (IM). The underlying rationale for IM™ training was that the processes of sequencing and coordinating movement patterns are based on an internal sense of rhythmicity and that this rhythmicity can be improved with practice to a metronome (Koomer et al. 2000).

The IM™ is a computer-based program that combines hand and foot tasks with an auditory guidance system. This produces a sequence of interactive exercises in which the participant strives to synchronise the task performance (either hand or foot movements) precisely with the auditory signals. For this intervention, the participant came twice a week for a period of 6 weeks and completed all his training sessions as the first part of his warm-up for the athletic training.

During IM™ training, the environment where the training was being held was regulated. On arrival, the participant was fitted with a hand and foot tap pad, with which to respond to the computer-based auditory signal (the computerised metronome beat) via headphones (IM™ mini headphones) that were fitted to the participant’s head. At the start of the programme, the participant was presented with specific tasks of tapping his hand, or foot, or both, in synchrony to the metronome beat. Computerised guide sounds provided feedback (three different feedback signals were sounded, a ‘too early’, ‘too late’ or a ‘so right on’) to assist the participant to fine-tune his movements to the beat. This was performed to provide corrective information, which was either reinforced or encouraged to be adjusted in subsequent tasks (Interactive Metronome 2007).

### Data analysis

The semi-quantitative analysis involved the digital video analysis of the athlete’s kinematic data, using video software (Dartfish ProSuite version 4.0.9.0), for the initial acceleration and the maximal running velocity phases during a 60-m sprint. Each variable (stride length and stride time) during each of these two phases of the sprint was calculated through the video analysis software and then compared over the two testing periods. Deductions from these data were made and discussed.

## Ethical consideration

With regard to ethical considerations, permission to conduct this study was obtained from Stellenbosch University’s Research Committee (ethics clearance number 233/2009). Permission from the participant was sought before commencing with the research. All information was treated with the strictest confidentiality, and the identity of the participant was protected. Personal information and names were not disclosed in the reporting of the findings.

## Results

**FIGURE 3:**
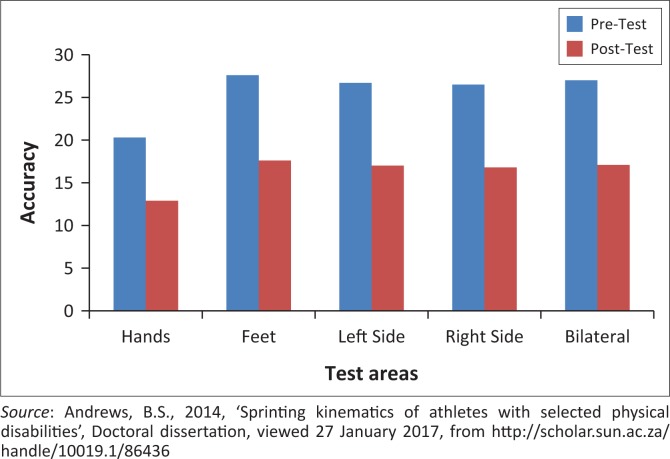
Accuracy improvements from the pretest evaluation to the post-test evaluation in the IM™ training.

A comparison between pretest and post-test scores using IM™ precision timing is presented in Figure 3. There was an improvement in the accuracy following the intervention period on tasks for the hands only, feet only, left side of the body, right side of the body as well as bilaterally. Table 1 presents the total summary for all task repetitions (3810) in the programme, showing a 36.6% improvement in the precise timing of movements after completing the intervention programme.

**TABLE 1:** Pretest and post-test adjustment values and improvement following Interactive Metronome™ training.

Total repetitions	Pretest	Post-test	Pretest to post-test improvement
3810	37.70	23.90	36.6%

*Source*: Andrews, B.S., 2014, ‘Sprinting kinematics of athletes with selected physical disabilities’, Doctoral dissertation, viewed 27 January 2017, from http://scholar.sun.ac.za/handle/10019.1/86436

The influence of IM™ training on the sprinting performance of this athlete can be identified within the differences between sprint kinematics reported for the pre-IM test and post-IM test. The learned effect is negated as he was an elite athlete and has completed countless sprints over his career, and therefore, any changes in the sprint kinematics could be attributed to the IM™ training. In order to make this comparison clear, the presentation of these data in the following figures is limited to the performance indicators of time, stride length and stride frequency (as discussed in the studies by Hay & Reid 1988).

**FIGURE 4:**
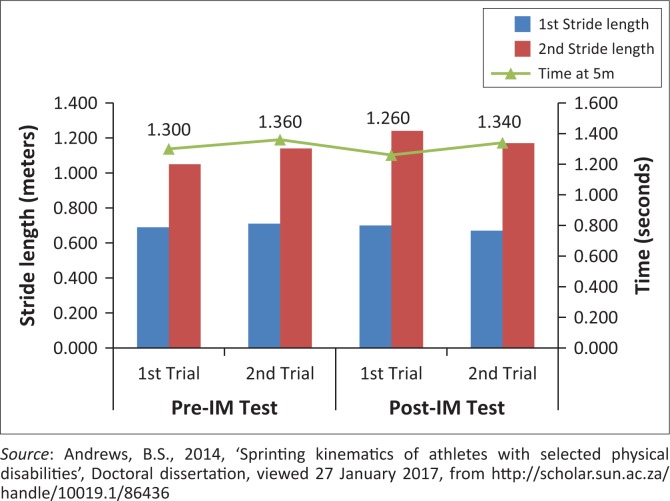
Effect of the first two stride lengths within the acceleration phase, on time at 5 m.

**FIGURE 5:**
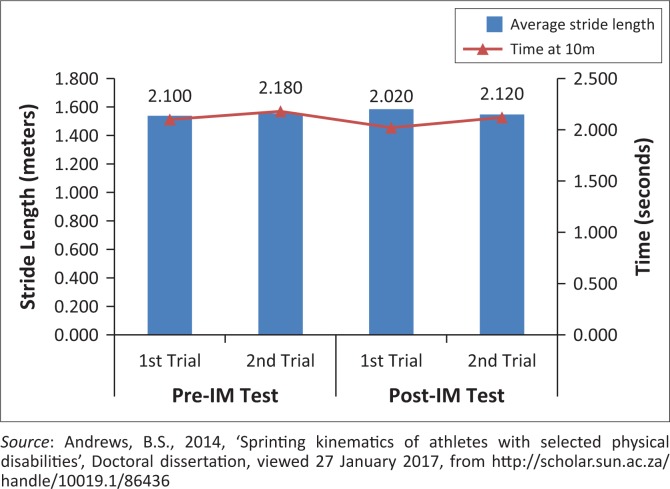
Effect of the average stride length within the acceleration phase, on time at 10 m.

**FIGURE 6:**
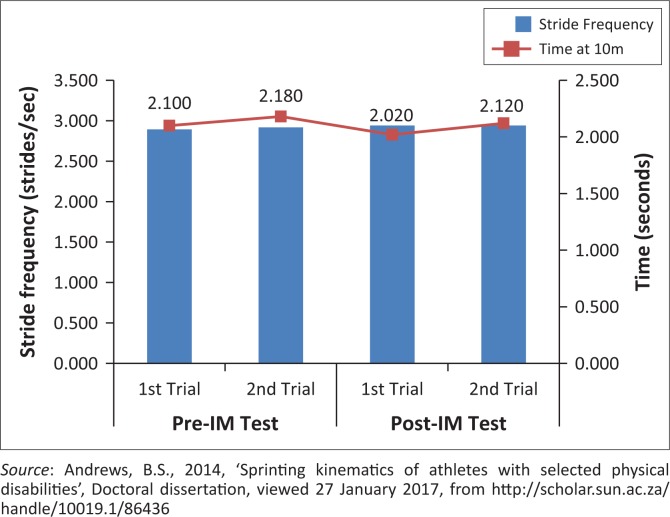
Effect of stride frequency within the acceleration phase, on time at 10 m.

The following trends can be noted after the IM™ training for three of the key performance indicators during the acceleration phase:

- Second stride length (always taken with his unaffected leg) appears to be more critical to sprint performance compared to the first stride length (always taken with prosthetic leg) for this athlete (Figure 4).
- Longer average stride lengths appear to be important for overall sprint performance (Figure 5).
- Stride frequency did not show to be an individually important factor in sprint performance (Figure 6).
- No consistent changes to the sprint kinematics following the IM™ training were seen. If IM™ training had any influence on the initial acceleration phase, it would be the slight increase in stride frequency. This increase, when coupled with an above average stride length, might have a positive impact on time and sprint performance.

**FIGURE 7:**
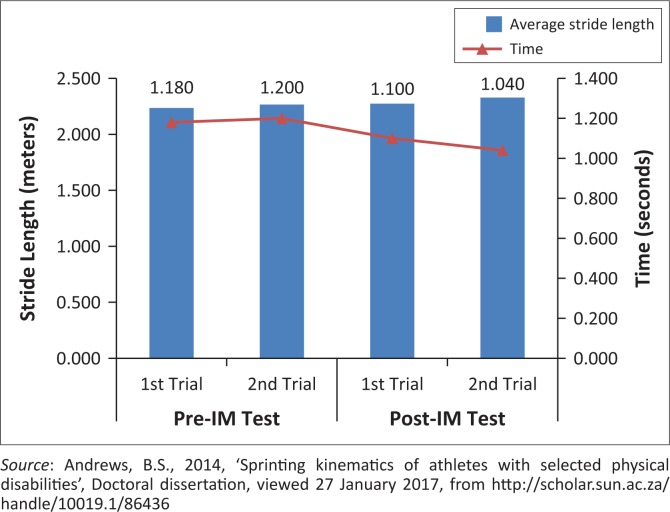
Effect of the average stride length during 10 m of the maximal running velocity phase, on time.

**FIGURE 8:**
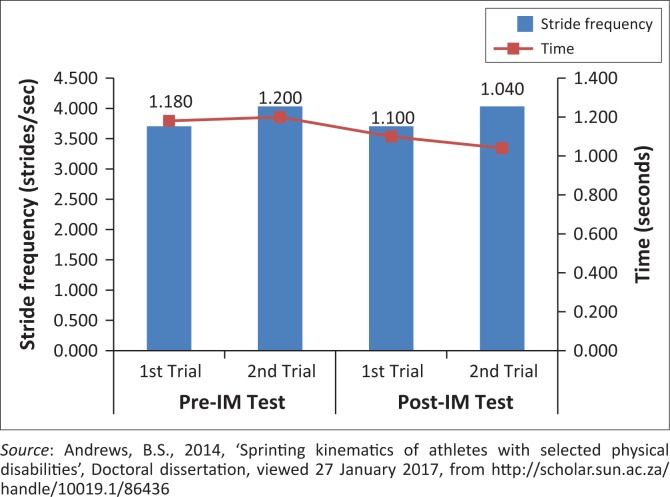
Effect of stride frequency during 10 m of the maximal running velocity phase, on time.

The following trends were noted after the IM™ training for three of the key performance indicators during the maximal running velocity phase:

- Longer average stride lengths appear to be important for overall sprint performance (Figure 7).
- Stride frequency did not show to be significantly important for sprint performance (Figure 8).
- If IM™ training had any affects, it was that of increasing stride length. This can be expected if this athlete’s coordination capacity improved, as was shown on the IM™ training. This would thus lead to an improved time and sprint performance.

## Discussion

Training programmes for timing and rhythmicity have been used in a variety of rehabilitation settings (Haas, Distenfeld & Axen 1986; Shumway-Cook & Woollacott 2007). However, studies showing the effect of timing and rhythmicity training on sport performance are limited (Sommer & Rönnqvist 2009). The present case study was conducted to describe the effect of SMT on motor timing on the sprint kinematics in an elite Paralympic below-knee amputee.

This research found that motor timing and rhythmicity of the athlete improved following an SMT programme, specifically IM™ training. This finding is supported by previous research (Koomer et al. 2000; Sommer & Rönnqvist 2009). However, this study found that, despite following the large improvements seen in the IM™ performance (a 36.6% improvement), only very minimal improvements were seen in the actual motor performance (observed in the kinematic data). This is in contrast to previous research by Libkuman, Otani and Steger (2002), who found that following an IM™ training programme produced significant improvements in timing and rhythmicity, which in turn lead to observable improvements in the motor performance.

Diamond (2003) noted that IM™ training contributes to a more efficient and consistent neural processing of muscle activation patterns. Furthermore, Myskja (2005) concluded that when movements become more rhythmically stable, a more optimal coordination of movement performance is achieved. This outcome would be advantageous to sprinters who are continuously striving for optimal efficiency and effectiveness in the execution of the coordination patterns that support the different phases of sprint performance (Sommer & Rönnqvist 2009).

Possible reasons why this has not been seen in the present case study might be related to the level of the athlete who in this instance was competing at the elite level (the Train to Compete stage) of the Long-Term Athlete Development model (Bhambhani & Higgs 2011). Furthermore, this athlete might have maximised his motor programming and movement patterns through many years of sprinting experience (Magill 2003). The optimal ages for skill development for able-bodied youth have been identified as 8–11 years of age for females and 9–13 years of age for males, based on peak height velocity estimates (Bhambhani & Higgs 2011). Lastly, the IM™ training might have been offered during the wrong training phase of this athlete’s periodisation programme (Bompa & Carrera 2005) and better results could be expected if the training had been conducted during this athlete’s ‘off-season’ (Bompa & Carrera 2005).

## Conclusion

A well-designed and carefully followed SMT programme, like that provided by the IM™, appears to produce improvements in timing and rhythmicity for participants. However, these improvements will not always be translated into sport-specific motor performance. Numerous other factors should also be considered, such as training phases and age or experience of the participants.

SMT programmes claim that they can assist in improving timing and rhythmicity for all participants at all levels. However, to have a consistent, reliable and significant impact on participants, SMT programmes should be followed by young participants in the more fundamental phases of their movement development.

## Recommendations

This study followed a single case. To make more meaningful recommendations a larger sample population would be ideal, but given the limited number of elite Paralympic athletes, this was not feasible. However, some deductions can be made from this study, and it is recommended that coaches have young athletes or players follow SMT programmes (like the IM™) during their skill development phase. This will improve their timing and rhythmic abilities, thereby improving their coordination and motor sequencing, which will provide these young athletes or players with the ability to optimise their sport-specific motor performance (Mastrokalou & Hatziharistos 2007).
